# Need of Rest

**Published:** 1885-03

**Authors:** C. H. Huyhes


### THE NEED OF REST.

The cause of much of the premature decrepitude and nerve degeneracy, and breakdown of our day, is in the many inventions man has devised whereby he robs himself of timely rest. The morning newspaper often read through before breakfast; the telephone in his house to call him at any and all times aside from his repose; the electric light to keep his brain unduly stimulated through the retinæ; the railroad and the sleeping coach which may keep him constantly on the rail (if he chooses to so travel) for continuous weeks without rest from the noisy and exhaustive cerebro-spinal concussions of this mode of travel; hasty meals and telegrams, and business, and nightmare sleep, all commingled, wither and wreck lives innumerable, which, under wiser management might end differently, and the needless noises of the city, the bells and steam whistles, howling hucksters, noisy street cars, yelling hoodlums, that make night hideous with soul jarring sounds, hasten the premature endings of useful lives. And when, superadded to all this unphysiological strain, we have the assault of a pestilence that poisons, like cholera, how much of resisting immunity can such overstrained and exhausted nerve force oppose to the invading foe?

If the epidemic comes, as it almost surely will next summer or fall, there should be a common understanding among physicians to demand as much rest as practicable for the people, and by comity among themselves, they should lighten each other's labors and no one should work continuously night and day.

It is not long after an epidemic comes before the long watching nurses and the tired, over-taxed doctors become its victims.

The lesson a pestilence teaches is not only cleanliness but temperance, and restful resisting vigor for the nervous system and the conservation of its powers, maintaining the functions of the body in the presence of a blood destroying and vitality depressing enemy. With the human organization, in a long contest with disease, the blood is the life, but if the nervous system has fortified itself, by ample rest and frugality and economy of expenditure ; and by freedom from overstrain and vicious indulgence, has established the habit of claiming and securing to recuperative use its own elements from the blood, it will be longer in yielding and longer still in perishing under the assaults of disease.—*C. H. Huyhes, M. D., in the Alienist and Neurologist.*

